# Hepatic artery infusion pump for nasopharyngeal carcinoma with liver metastasis

**DOI:** 10.1007/s10585-019-10015-0

**Published:** 2019-12-20

**Authors:** Changli Peng, Chunhui Zhou, Gang Li, Haiping Li, Liangrong Shi

**Affiliations:** Radiological Intervention Center, Department of Radiology, Xiangya Hospital, Central South University, 87 Xiangya Road, Changsha, 410008 Hunan China

**Keywords:** Hepatic artery infusion, Nasopharyngeal carcinoma, Liver metastasis

## Abstract

To evaluate the benefits and risks of hepatic artery infusion (HAI) gemcitabine and floxuridine (FUDR) in patients with nasopharyngeal carcinoma liver metastases. HAI catheter systems were implanted under the guide of digital subtract angiography (DSA) in 16 patients with unresectable nasopharyngeal carcinoma liver metastases. HAI gemcitabine and FUDR in combination with radiotherapy and systemic chemotherapy were delivered. Disease control rate (DCR) of intrahepatic lesions is 100%, objective response rate (ORR) of intrahepatic lesions is 87.5%, including 4 patients (25%) with complete response (CR), 10 patients (62.5%) with partial response (PR) and 2 patients (12.5%) with stable disease (SD). The median overall survival (mOS) was 30 months. There was no significant difference between patients with < 9 intrahepatic lesions and patients with ≥ 9 intrahepatic lesions (31 months vs. 24 months, *P* = 0.562). Patients without extrahepatic metastases has longer survival than patients with extrahepatic metastases (31 months vs. 17 months, *P* = 0.005). In all 72 cycles of HAI, the main grade 3/4 toxicities related to HAI include: leukopenia occur in 8 cycles (11.1%), thrombocytopenia in 5 cycles (6.9%), AST/ALT elevation in 12 cycles (16.7). Catheter related complications occurred in 2 patients (12.5%). HAI gemcitabine and FUDR is effective to improve DCR of intrahepatic lesions and prolong mOS for patients with nasopharyngeal carcinoma liver metastases, and is associated with a relative low rate of toxicity.

## Introduction

Nasopharyngeal carcinoma (NPC) is a common head and neck malignancy, with a high prevalence in South-East Asia, particularly in southern China [1]. It is estimated that in China, NPC accounts for about 1.34% of all new cancer cases per year and 1.03% of all cancer deaths per year, with a prevalence of 3.16/100,000 and 1.53/100,00 respectively [2]. Unlike other head and neck cancers, NPC presents with high incidence of locoregional recurrence or distant metastases [3], which is considered as the predominant cause of mortality [4]. The most common metastatic sites are bone, liver and lung [5]. Liver metastases of NPC often present as multifocal nodules, and unfortunately has worse prognosis compared with metastasis to bone or to lung, with median overall survival of 3–5 months [6, 7]. According to NCCN guidelines, treatment options for metastatic NPC include Clinical trials (preferred), Platinum-based combination chemotherapy or concurrent chemo/radiotherapy [8], but the clinical outcome is still limited. Thus how to improve the prognosis of NPC patients with liver metastasis remains a big challenge.

It is reported that perioperative hepatic arterial infusion pump chemotherapy is associated with longer survival after resection of colorectal liver metastases [9]. Previous work by our team showed that initial hepatic artery infusion (HAI) and systemic chemotherapy is helpful for colorectal cancer patients with liver metastases to obtain a high resection rate [10]. Hepatic-directed therapy is recommended for some liver-predominant disease in patients with neuroendocrine tumors of the pancreas [11]. Here, we use this HAI regime in NPC patients with liver-predominant metastases, aiming to verify its effectiveness and safety.

## Patients and methods

### Patients selection

From January 2011 to December 2017, we treated 16 consecutive patients with NPC liver-predominant metastases. The following clinical data were collected: age, gender, performance status, UICC stage, pathological type, type of systemic chemotherapy, volume proportion of involved liver, number of intrahepatic lesions, status of portal vein thrombus, extrahepatic metastases, Child–Pugh class of liver function, objective response status of intrahepatic lesions (assessed with CT-enhanced scan according to RECIST criteria), survival in months from time of catheter implantation. The treatment protocol for each patient was discussed and determined by a multi-discipline treatment (MDT) in our hospital, which includes medical oncologist, otolaryngologist, radiologist and interventional specialist. Before the initial treatment, an informed consensus was achieved, which was approved by the Ethics Committee of our hospital.

### HAI catheter system implantation

The infusion catheter and injection port (Celsite, B. Braun, Chasseneuil, France) was implanted as previously described by our team and other authors [10, 12, 13]. The key points of the technique include: (i) Computed tomography angiography before operation to assess the anatomy of hepatic artery and indication for HAI therapy; (ii) Under the guide of digital subtraction angiography (DSA), the Seldinger technique was used to establish access to right femoral artery. (iii) Angiography of celiac trunk and superior mesenteric artery was performed to confirm the anatomy of hepatic artery; (iv) Embolization of the gastro-duodenal artery (GDA), right gastric artery, and, if necessary, left gas-tric artery or dorsal pancreatic artery with metal coils (Tornade, Cook, Bloomington, IL, USA) to prevent extra-hepatic infusion of chemotherapeutic agents and resulting gastro-duodenal injury; (v) Side-hole infusion catheter was inserted in GDA or peripheral branch of hepatic artery, followed by the corresponding vessels embolized using the same coils as mentioned above to fix the infusion catheter, with the side-hole positioned in the common hepatic artery. Thus the chemotherapeutic agents could infuse the entire liver from the side-hole; (vi) the proximal end of the infusion catheter was connected to an injection port and the device was implanted in a subcutaneous pocket in the right inner thigh. After the administration of chemotherapeutic agents, the implanted port and indwelling catheter system were flushed and filled with 2 mL of heparin solution (1000 IU/mL).

### HAI therapy and systemic chemotherapy

All patients received a 3-week cycle of HAI the next day after catheter implantation. The HAI therapy was performed on day 1, 8: Gemcitabine 1 g/m^2^ for 30 min, followed by a blended solution which comprised floxuridine (FUDR) at 0.15 mg/kg/day, dexamethasone (DXM) at 1 mg/m^2^/day, low molecular heparin 3200U and saline, lasted for 7 days continuously. This type of HAI regime was accomplished by a 14-day infusor (Baxter). Standard treatment of NPC, including radiotherapy and chemotherapy (induction chemotherapy, concurrent chemotherapy and adjuvant chemotherapy) was performed as necessary. Dose adjustment was made in the event of toxicity, assessed according to National Cancer Institute-Common Terminology Criteria for Adverse Events (NCI-CTCAE) version 3.0. The HAI therapy was stopped if serious technical catheter-related problems, progression of intrahepatic disease or excessive toxicity occurred. Response to chemotherapy was assessed every 2 HAI cycles or when necessary by spiral-CT scan according to the RECIST criteria [14]. Epigastric pain prompted workup with an upper gastrointestinal endoscopy. If an ulcer or gastro-duodenitis was documented, HAI therapy was held for 1 month to allow healing and the dosage of FUDR and DXM was reduced by 50% in subsequent therapies.

### Statistical analysis

The main endpoint of the study was objective response rate (ORR). Overall survival (OS) was defined as the time from the date of catheter implantation to the date of death or the date of the last follow-up, median survival time (mOS) was defined as the time from the date of catheter implantation to the date 50% of individuals is alive. The 3-year survival rates were estimated by using the Life Table method. The survival analysis was performed by using the log-rank test.

## Results

### Patients’ baseline of characteristics

Sixteen patients (13 males and 3 females) were included in the study, with the median age of 56yrs (30–78 years). All of them scored 0–2 in ECOG (Eastern Cooperative Oncology Group) score standard, staged II–IV in UICC (The Union of International Cancer Control) stage system. The pathology type of NPC was confirmed by biopsy, comprised Non-keratinized differentiated type (n = 2) and Non-keratinized undifferentiated type (n = 14). Systemic chemotherapy was performed in all patients, which included induction chemotherapy, concurrent chemotherapy and adjuvant chemotherapy. All of the baseline of characteristics are summarized in Table 1.

**Table 1:** Baseline of characteristics (n = 16)

Characteristics	n (%)
Age (year)	
Median	56
Range	30–78
Gender	
Male	13 (81.3)
Female	3 (18.7)
Performance status	
0	5 (31.25)
1	10 (62.5)
2	1 (6.25)
UICC stage^a^	
II	3 (18.7)
III	11 (68.8)
IV	2 (12.5)
Pathological type	
Non-keratinized differentiated	2 (12.5)
Non-keratinized undifferentiated	14 (87.5)
Chemotherapy	
Induction	4 (25.0)
Concurrent	14 (87.5)
Adjuvant	6 (37.5)

*UICC* The Union of International Cancer Control, *ECOG* Eastern Cooperative Oncology Group

^a^Patients’ staging status according to UICC/AJCC Cancer Staging Manual (Eighth Edition) at initial diagnosis. Two patients presented synchronous liver metastasis and were defined as stage IV, the other 14 patients presented metachronous liver metastasis and were defined as stage II or stage III according to their status of T criteria and N criteria

### Baseline information of hepatic metastases

The status of hepatic metastases, including synchronous hepatic metastases at initial diagnosis of NPC, and disease progresses to hepatic metastases, was assessed by proportion of hepatic involvement, lobular involvement, number of hepatic lesions, existence of extrahepatic metastases, and baseline level of albumin. See Table 2. Notice that majority of patients presents bilobar involvement (n = 13, 81.3%), multifocal metastases (9 patients with intrahepatic lesions ≥ 9, 56.2%). No patient is indicated for metastases resection (diffused intrahepatic lesions or bilobar involvement).

**Table 2:** Baseline of hepatic metastases (n = 16)

Characteristics	n (%)
Hepatic involvement	
< 25%	3 (18.8)
25–75%	11 (68.7)
> 75%	2 (12.5)
Lobulor involvement	
Bilobar	13 (81.3)
Unilobar	3 (18.8)
Number of lesion	
< 5	2 (12.5)
5–9	5 (31.3)
> 9	9 (56.2)
Extrahepatic metastasis	
No	9 (56.2)
Lung	3 (18.8)
Bone	5 (31.3)
Baseline of albumin (mg/mL)	
≥ 35	9 (56.2)
< 35	7 (43.8)
Portal vein thrombus	0
Child–Pugh class	
A	10 (62.5)
B	6 (37.5)

### Response

**Fig. 1:**
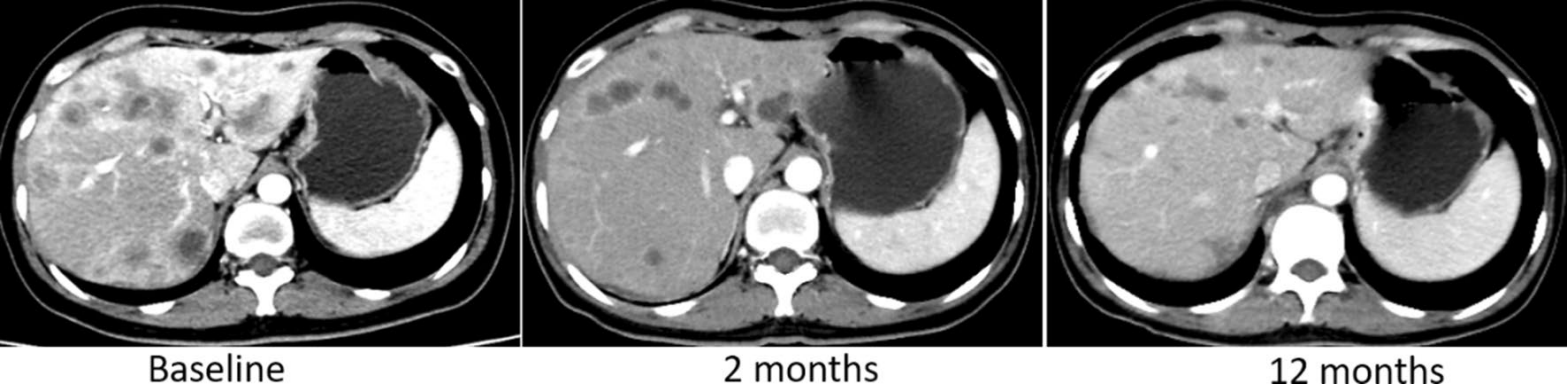
A typical patient’s CT imaging of liver. CT image at baseline showed extensive intra-hepatic lesions. CT image showed the intra-hepatic lesions shrinking after 2 months of HAI regime. Intra-hepatic lesions further shrank after 12 months of HAI regime. This patient was judged as exhibiting a partial response

Intrahepatic lesions were assessed every 2 HAI cycle or when necessary, with enhanced spiral-CT scan according to RECIST criteria (version 1.1). 4 patients (25%) achieved complete response (CR), 10 patients (62.5%) achieved partial response (PR), 2 patients (12.5%) achieved stable disease (SD), none with local progressive disease (PD). That is to say, the objective response rate (ORR) is 87.5% (CR + PR), disease control rate (DCR) of intrahepatic lesions is 100% (CR + PR + SD), but 3 patients (18.8%) had extrahepatic progression during the trial treatment. See Table 3. Figure 1 demonstrates a typical CT imaging of one patient.

**Table 3:** No. of HAI cycle, response and catheter related complications for individual patient

Case	HAI cycles	Intrahepatic response	Extrahepatic progression	Cather related complications
1	5	PR	No	No
2	6	CR	No	No
3	3	SD	Yes	No
4	8	PR	No	No
5	3	PR	No	Yes
6	4	PR	No	No
7	4	CR	No	No
8	2	PR	Yes	No
9	6	PR	No	No
10	2	PR	Yes	No
11	6	CR	No	No
12	7	PR	No	No
13	2	PR	No	Yes
14	4	SD	No	No
15	6	CR	No	No
16	4	PR	No	No

### Survival analysis

**Fig. 2:**
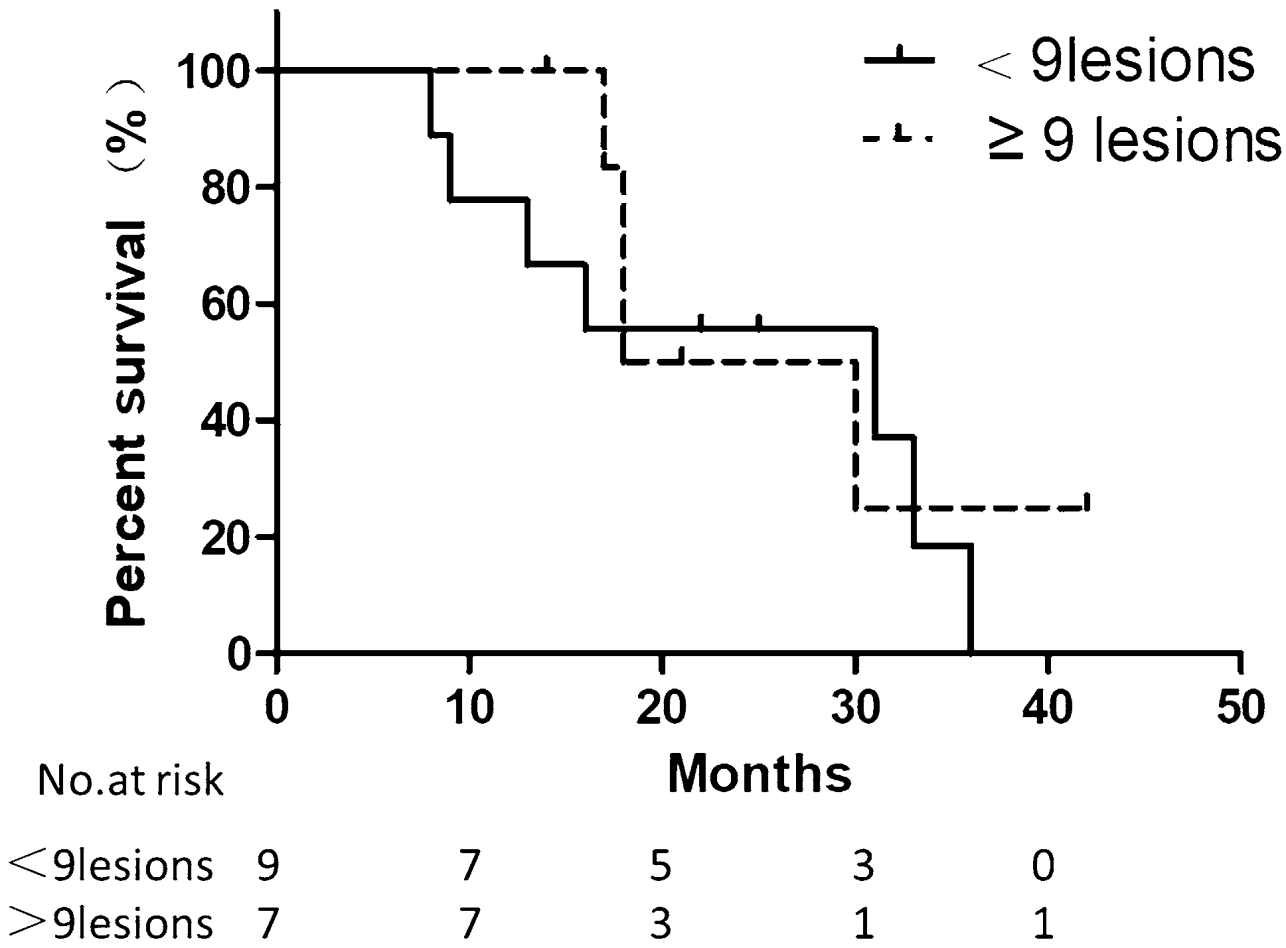
Kaplan–Meier estimates of OS for patients grouped by number of intra-hepatic lesions. Median overall survival (mOS) for patients with < 9 intra-hepatic lesions is 31 months, while mOS for patients with ≥ 9 intra-hepatic lesions is 24 months, calculated from the date of catheter implantation. (P = 0.562, log-rank test)

**Fig. 3:**
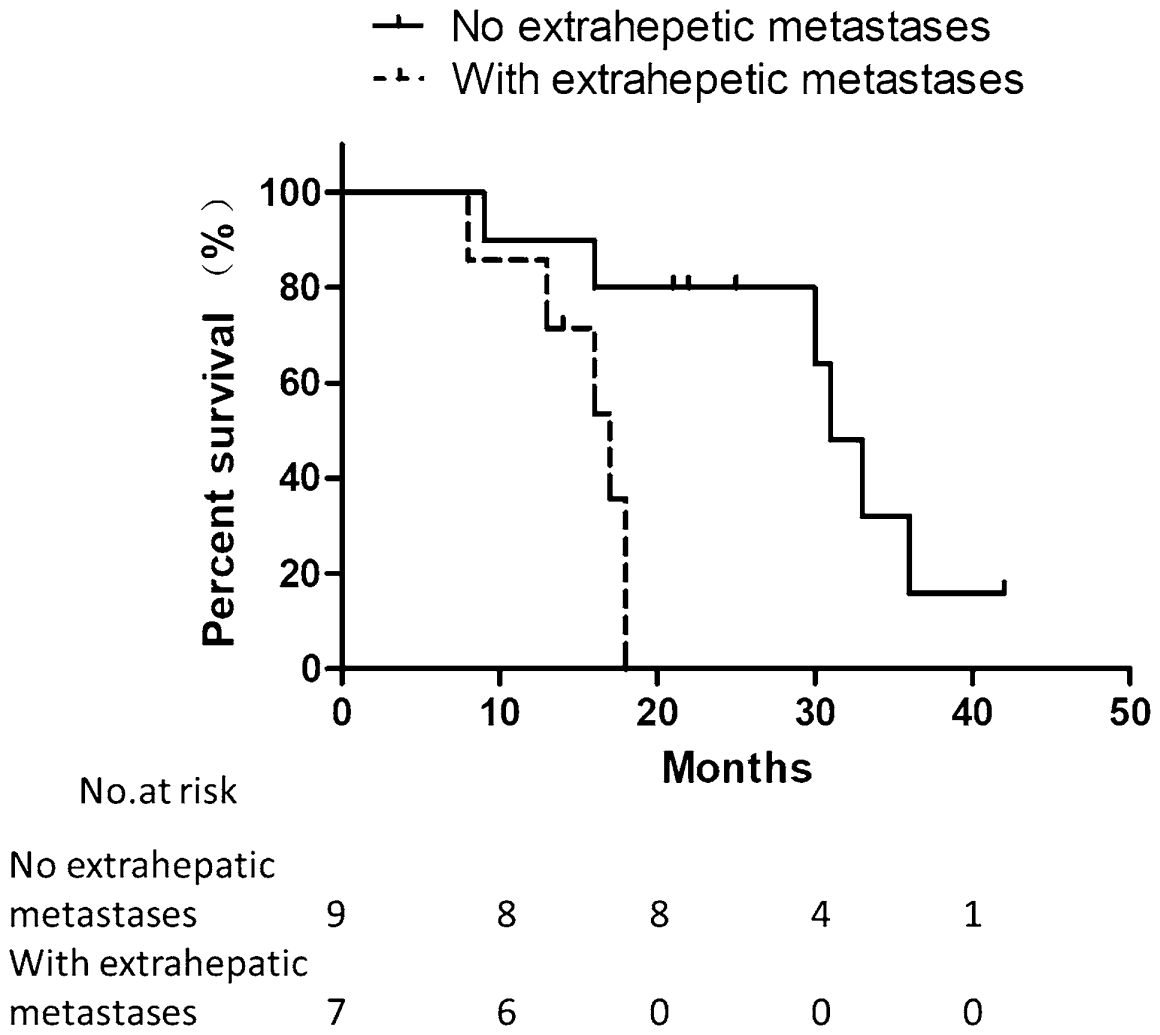
Kaplan–Meier estimates of OS for patients grouped by existence of extra-hepatic metastases. Median overall survival (mOS) for patients without extra-hepatic lesions is 31 months, while mOS for patients with extra-hepatic lesions is 17 months, calculated from the date of catheter implantation. (P = 0.005, log-rank test)

The median follow up was 31 months (range 8 to 42 months). On May 31, 2019, 5 patients were still alive. The median overall survival (mOS) was 30 months. The mOS was 31 months and 24 months for patients with < 9 intrahepatic lesions and ≥ 9 lesions, respectively (*P* = 0.562). As grouped by the existence of extrahepatic metastases, patients with extrahepatic metastases had a median survival of 17 months, while patients without extrahepatic metastases had a median survival of 31 months, there was analytical significance (*P* = 0.005). See Figs. 2 and 3.

### Side effects and adverse events

In summary, all the 16 patients underwent a total number of 72 cycles of HAI therapy as showed in Table 3, and the number of cycles in which any side effect or adverse event could be observed or detected was accumulated. The most common toxicities include: leukopenia occur in 36 cycles (50%) including grade 1/2 in 28 (38.9%) and grade 3/4 in 8 (11.1%), thrombocytopenia in 25 (36.1%) including grade 1/2 in 21 (29.2%) and grade 3/4 in 5 (6.9%), AST/ALT elevation in 8 (66.7%) including grade 1/2 in 36 (50.0%) and grade 3/4 in 12 (16.7%). In addition, grade 3/4 abdominal pain occurred in 3 cycles (4.2). No grade 3/4 hyperbilirubinemia occurred. The main toxicities was showed by cycles as listed in Table 4. Catheter related complications occurred in 2 patients (12.5%) including catheter occlusion in 1 patient after 2 cycles of HAI and catheter displacement occurred in 1 patient after 3 cycles (Table 3). HAI was discontinued in these patients. No long-term complication such as biliary toxicity and hepatic artery occlusion was observed in the study.

**Table 4:** Most common toxicities by cycles [n (%)]

Toxicities	Grades 1/2	Grades 3/4
	n (%)	n (%)
Hematological		
Leukopenia	28 (38.9)	8 (11.1)
Neutropenia	26 (36.1)	6 (8.3)
Anemia	14 (19.4)	2 (2.8)
Thrombocytopenia	21 (29.2)	5 (6.9)
AST/ALT elevation	36 (50.0)	12 (16.7)
Hyperbilirubinemia	9 (12.5)	0
Gastrointestinal		
Anorexia	8 (11.1)	0
Nausea	17 (23.6)	0
Vomiting	14 (19.4)	2 (2.8)
Diarrhea	15 (20.8)	3 (4.2)
Fatigue	20 (27.8)	2 (2.8)
Abdominal pain	15 (20.8)	3 (4.2)

## Discussion

Several studies have been shown that HAI is effective in improving hepatic response rates in colorectal cancer liver metastasis. In the present study, we treated 16 consecutive NPC patients with predominant liver metastasis using HAI of Gemcitabine and FUDR. Our data show that the ORR was 87.5% and the mOS was 30 months. The majority (223/266, 83.8%) of toxicities and adverse events was judged on Grades1/2.

About one-third of patients with NPC suffer of distant metastases and resultant treatment failure [15]. Liver metastasis is the second common metastasis site, which accounts for 30% of such cases, following bone metastasis (70%) and followed by lung metastasis (18%) [16], but has the worst prognosis (with reported mOS of 3–5 months) [6, 7]. Thus it is of significance to explore new therapeutic strategy.

HAI has been proven both by our previous work and other authors to be helpful in some patients [9, 10]. It is also accepted as first-line treatment for unresectable colorectal cancer liver metastases [17]. Here we administered HAI for patients with NPC liver metastasis. Given that in our case series, the majority of patients suffered of unresectable dominant liver involvement (defined as liver involvement as the dominant site of metastasis, hepatic metastases constitute ≥ 50% of all tumor burden [18]): 13 patients (81.2%) had hepatic involvement > 25%, 13 patients (81.2%) had bilobar involvement, 14 patients (87.5%) had number of intrahepatic lesions > 5 (among whom 9 patients with number of intrahepatic lesions > 9), the ORR and DCR of intrahepatic lesions as well as survival analysis is encouraging.

Nowadays treatment options for metastatic NPC include Clinical trials, Platinum-based combination chemotherapy or concurrent chemo/radiotherapy [8], but prognosis is still poor. Some investigators have reported systemic monochemotherapy for metastatic NPC with ORR range from 28 to 48% [19-21], and some polychemotherapy protocols with ORR range from 42.7 to 73% [20, 22, 23]. It seems that patients in current study probably have survival benefit. Theoretically, controlling intrahepatic lesions decreases the total tumor burden, then yields survival benefit. It is reported that elimination liver metastasis of nasopharyngeal carcinoma might improve overall survival [24], which might have something in common with current study as respect to the underlying mechanism of survival benefit.

In current study, chemotherapy agents are administered through intra-arterial approach, directly targeting intrahepatic lesions. Especially for FUDR, the low-dose and long-period infusion may provide higher intact drug concentration in liver and minimal systemic toxicity [25].

Besides HAI, patients in current study also underwent comprehensive therapy to primary lesion. This combined treatment modality supplies control power both of the primary tumor and of the liver metastases, which may contribute to survival benefit. But there was still 3 patients (18.8%) who had extra-hepatic progression during the trial treatment, indicating the necessity to explore more effective treatment strategies.

Several side effects and adverse events could be observed or detected in current study, including (i) hematological toxicities such as leukopenia, neutropenia, anemia, thrombocytopenia; (ii) AST/ALT elevation; (iii) hyperbilirubinemia; (iv) gastrointestinal toxicities such as anorexia, nausea, vomiting, diarrhoea; (v) fatigue; (vi) abdominal pain. For all the toxicities mentioned above, the proportion for grades 1/2 toxicities is obviously higher than that for grades 3/4 toxicities. Some toxicities resulted from the same underlying mechanism as systemic chemotherapy. AST/ALT elevation appeared in 48 cycles (66.7%), but the majority of them (n = 36, 50%) was on grades 1/2, while the minority (n = 12, 16.7%) was on grades 3/4. This might reveal the safety of this strategy for liver function. Some authors reported biliary toxicity of HAI with FUDR [25]. In current study, reversible Grade1/2 Hyperbilirubinemia is observes in 9(12.5%) cycles, with no evidence of long-term biliary damage, probably because of adding DXM into FUDR solution. Technically, the gastrointestinal toxicities might derive from extra-hepatic infusion of FUDR to stomach or duodenum, so the main branch vessels from the associated arteries (e.g. Hepatic artery or GDA) were routinely embolized in operation, but this still could not be completely avoided. Fortunately, most patients with grades 3/4 toxicities continued HAI therapy, even though some needed dose reduction or gap prolongation. If indwelled directly in hepatic artery, the dislodgement of the infusion catheter tip may result in vessel damage, occlusion or aneurysm, finally lead to failure of the whole infusion system. Here we fixed the side-hole infusion catheter in GDA or in the relatively smaller branch of hepatic artery, positioned the side-hole in common hepatic artery, thus effectively avoided the potential risk and guaranteed the unidirectional infusion to liver. Other HAI related complications, such as bleeding, thrombosis or infection are not observed in these patients. In our opinion, these data, to some extent, could prove the safety of our HAI strategy.

This study has several limitations. We performed HAI for NPC patients with liver-predominant metastasis, whose intrahepatic disease status is more advanced compared to those who do not undergo HAI. As a retrospective study with small sample size, it lacks a control group with matched status of NPC liver metastases. The improvement of DCR of intra-hepatic lesions and survival benefit needs to be further investigated and confirmed by multi-center randomized study.

In conclusion, we introduced a new treatment strategy for NPC liver metastases, which may improve DCR of intra-hepatic lesions and prolong mOS. It can be considered as an option for suitable patients.
